# Facilitated Uptake of a Bioactive Metabolite of Maritime Pine Bark Extract (Pycnogenol) into Human Erythrocytes

**DOI:** 10.1371/journal.pone.0063197

**Published:** 2013-04-30

**Authors:** Max Kurlbaum, Melanie Mülek, Petra Högger

**Affiliations:** Universität Würzburg, Institut für Pharmazie und Lebensmittelchemie, Würzburg, Germany; East Carolina University, United States of America

## Abstract

Many plant secondary metabolites exhibit some degree of biological activity in humans. It is a common observation that individual plant-derived compounds *in vivo* are present in the nanomolar concentration range at which they usually fail to display measurable activity *in vitro*. While it is debatable that compounds detected in plasma are not the key effectors of bioactivity, an alternative hypothesis may take into consideration that measurable concentrations also reside in compartments other than plasma. We analysed the binding of constituents and the metabolite δ-(3,4-dihydroxy-phenyl)-γ-valerolactone (M1), that had been previously detected in plasma samples of human consumers of pine bark extract Pycnogenol, to human erythrocytes. We found that caffeic acid, taxifolin, and ferulic acid passively bind to red blood cells, but only the bioactive metabolite M1 revealed pronounced accumulation. The partitioning of M1 into erythrocytes was significantly diminished at higher concentrations of M1 and in the presence of glucose, suggesting a facilitated transport of M1 via GLUT-1 transporter. This concept was further supported by structural similarities between the natural substrate α-D-glucose and the S-isomer of M1. After cellular uptake, M1 underwent further metabolism by conjugation with glutathione. We present strong indication for a transporter-mediated accumulation of a flavonoid metabolite in human erythrocytes and subsequent formation of a novel glutathione adduct. The physiologic role of the adduct remains to be elucidated.

## Introduction

Maritime pine bark extract is monographed in the United States Pharmacopeia (USP) as a dietary supplement [1]. A standardized pine bark extract that conforms with this monograph is derived from of *Pinus pinaster, Ait.,* (Pycnogenol®, Horphag Research Ltd., UK). Procyanidins consisting of catechin and epicatechin moieties of varying chain lengths represent approximately 65–75% of this extract [2], [3]. Other constituents are polyphenolic monomers, phenolic or cinnamic acids and their glycosides. Pycnogenol® revealed diverse pharmacological actions in human trials, e.g. anti-inflammatory and cardiovascular effects [3], [4]. So far there is still limited information on which compound(s) of the complex extract are mainly responsible for the documented bioefficacy.

One critical point with plant extracts is always the bioavailability of their constituents. Typically only low plasma concentrations are found after ingestion of dietary polyphenols or plant extracts [5]. In a pharmacokinetic study with single and multiple doses of Pycnogenol® we detected catechin, caffeic acid, ferulic acid, and taxifolin in the nanomolar range in the plasma of human volunteers [6]. We also found a maritime pine bark metabolite, δ-(3,4-dihydroxy-phenyl)-γ-valerolactone (M1), in the plasma samples. This metabolite is no constituent of the extract, but is generated *in vivo* from the procyanidins' catechin units through multiple step reactions. This metabolite M1 has been also found in urine samples [7]–[10].

We previously investigated the bioactivity of M1 and discovered pronounced antioxidant activity as well as inhibitory effects upon various matrix metalloproteinases [11] which was consistent with the reported anti-inflammatory effects of the extract. However, again the plasma concentrations of M1 were only in the nanomolar range [6] which was too low to induce any effects *in vitro*. Though it is possible that M1 is not the main mediator of maritime pine bark extract's bioefficacy it is also conceivable that plasma is not the only compartment where M1 is present *in vivo*. Previously, significantly higher recoveries of quercetin and resveratrol were reported from whole blood compared to plasma which suggests that the polyphenols are also distributed into the cellular blood fraction [12]. Recently we observed pronounced uptake of M1 into endothelial cells and monocytes/macrophages *in vitro*. The uptake was decreased by phloretin, suggesting a facilitated transport mechanism [13].

Though the partitioning of compounds into red blood cells has received less attention than the plasma protein binding, erythrocytes constitute a significant compartment for distribution [14], [15]. We recently analyzed the plasma protein binding of various maritime pine bark polyphenols and observed pronounced differences in the binding tendency [16]. While catechin and taxifolin displayed protein binding close to 100%, low binding around 30% was seen for M1 and its structurally related metabolite M2 (δ-(3-methoxy-4-hydroxy-phenyl)-γ-valerolactone). The purpose of the present investigation was to analyse the binding of selected Pycnogenol® constituents and the metabolite M1 to human erythrocytes to gain further insight into the disposition of these compounds.

## Materials and Methods

### Chemicals and reagents

Ferulic acid, (±)-taxifolin, caffeic acid, *p*-coumaric acid, glutathione, glutathione-S-transferase (EC 2.5.1.18), phloretin, and 2,2′-azobis(2-amino propane (AAPH), cytochalasin B from *Drechslera dematioidea,* D (+)-glucose, ethylene glycol-bis(2-aminoethylether)-*N,N,N′,N′*-tetraacetic acid (EGTA), were all obtained from Sigma-Aldrich (Taufkirchen, Germany). 4-(2-Hydroxyethyl)piperazine-1-ethanesulfonic acid (HEPES) was purchased from Gerbu (Wieblingen, Germany). The metabolite M1 (δ-(3,4-dihydroxy-phenyl)-γ-valerolactone) was synthesized by M. Rappold as part of his diploma thesis [17]. Methanol (HPLC grade) was obtained from Merck (Darmstadt, Germany), acetonitrile (HPLC grade) was from Fisher Scientific (Schwerte, Germany). Ultrapure Milli-Q water was used for all aqueous solutions. All other chemicals were purchased from Sigma-Aldrich.

### Buffers and human plasma/erythrocytes

The phosphate buffered saline (PBS, pH 7.4) consisted of 137 mM NaCl, 2.7 mM KCl, 8.1 mM Na_2_HPO_4_ and 1.5 mM KH_2_PO_4_. In case of incubation with erythrocytes the PBS buffer was supplemented with 0.1% (m/V) glucose. The buffer used in the AAPH assay (pH 7.4) consisted of 150 mM NaCl, 8.1 mM Na_2_HPO_4_ and 1.9 mM NaH_2_PO_4_ and 0.05% (m/V) glucose.

Human plasma and packed red blood cells were obtained from the blood banks of the University Hospital of Würzburg and of the Bayerisches Rotes Kreuz, München, Germany.

### Distribution of a polyphenol mixture between human plasma and erythrocytes

Packed red blood cells were washed twice with a threefold volume of cold PBS buffer (8°C) and centrifuged for 5 min at 952 *g* (10°C). Cells were weighted and assuming a density of 1.114 g/mL [18] 1.67 g were mixed with 2.0 mL plasma to obtain a hematocrit value of 0.43. The plasma contained a mixture of 1.3 µM caffeic acid, 80 µM ferulic acid, 6 µM taxifolin and 6 µM metabolite M1. The chosen concentrations were based on analytical considerations and previously also used for determination of plasma protein binding of these compounds [16]. In parallel a control was prepared containing the polyphenols in 3.5 mL plasma without erythrocytes. The tubes were incubated at 37°C and samples of 250 µL erythrocytes/plasma or plasma, respectively, were drawn and centrifuged at 952 *g* for 5 min (10°C). 100 µL of the supernatant was mixed with 10 µL of the internal standard *p*-cumaric acid, 40 µL 0.5 M hydrochloric acid and 130 µL methanol. After centrifugation at 14,000 *g* for 15 min (4°C) 20 µL were directly injected into the HPLC. In case of inhibition experiments the erythrocytes were pre-incubated with 600 µM phloretin (445 mL of a stock solution of 20 mg phloretin in 10 mL PBS buffer containing 0.01% DMSO) for 15 min and the samples were subsequently treated as described above. The erythrocyte/plasma partitioning ratio of the compounds was determined based on the peak area ratios to the internal standard as described by Yu et al. [19].

To ensure the cell vitality the percentage of haemolysed erythrocytes was determined according to Salauze [20] by photometric measurement of haemoglobin in plasma at 450 nm. Plasma was used as blank and samples of the erythocytes/plasma incubation were compared to completely haemolysed erythrocytes obtained after one freeze-thaw cycle (−80°C). The % haemolysis was calculated from the absorption of the cell supernatant in relation to the absorption of the totally haemolysed sample. In all experiments the percentage of haemolysed erythrocytes was below 3% over the whole experimental period.

### Uptake of M1 into human erythrocytes

Packed red blood cells were incubated with a threefold volume of PBS buffer with 100 mM D-glucose for 30 min at 37°C and centrifuged for 5 min at 2,000 *g* temperated to 4°C (Mikrofuge 22 R, Beckmann Coulter^TM^, Krefeld, Germany). Thereafter these cell pellets were washed twice with the threefold volume of cold PBS buffer (4°C) containing 100 mM D-glucose and centrifuged for 5 min at 2,000 *g* (4°C). 43 µL of these packed glucose-saturated cells were mixed with PBS buffer to obtain a hematocrit of 0.043. The cells were subsequently incubated with various concentrations of M1 (0.3–10 µM) for 1 min by rocking (Mini Rocker MR-1, Hartenstein, Würzburg, Germany) in closed reaction tubes (Eppendorf, Hamburg, Germany) at room temperature. In parallel control experiments were carried out accordingly for each variable without cells to monitor the stability of M1 during the experimental procedures. Similar to the procedure described by Leitch and Carruthers [21] the reaction was interrupted by adding a cold stop solution (4°C) containing 150 mM KCl, 5 mM MgCl_2_, 5 mM EGTA, 5 mM HEPES, 20 µM cytochalasin B and 200 µM phloretin in PBS buffer (pH 7.4), followed by a centrifugation of the cell preparations and matched controls for 5 min at 2,000 *g* (4°C). The supernatants were harvested and immediately analyzed by HPLC.

In case of competition experiments the erythrocytes were glucose-deprived. Cells were washed twice with the threefold volume of cold PBS buffer (4°C) without D-glucose and centrifuged for 5 min at 2,000 *g* (4°C). After incubating the cells with a threefold volume of PBS buffer (without D-glucose) for 30 min at 37°C and centrifugation for 5 min at 2,000 *g* (4°C), they were washed twice with the threefold amount of cold PBS buffer (4°C; without D-glucose) again and centrifuged for 5 min at 2,000 *g* (4°C). Subsequently, samples and controls were treated as described above, but this time in addition with 100 mM D-glucose to the various concentrations of M1 (0.3–10 µM). The erythrocyte/buffer partitioning ratio, or rather distribution coefficient, of M1 was determined based on the peak area ratios to the internal standard as described by Yu et al. [19].

In order to ensure equivalent cell counts in the experiments with glucose-saturated and glucose-deprived cells (competition experiments) the UV/VIS-absorption of free hemoglobin was measured in the supernatant after cell lysis. Therefore, the incubation mixtures with a hematocrit of 0.043 were prepared exactly as described above. In case of experiments with glucose-saturated erythrocytes (without D-glucose in the subsequent incubation) 43 µL of these cells were mixed with 957 µL PBS buffer. Simultaneously, 43 µL of glucose-deprived cells prepared for the competition experiments (with D-glucose in the subsequent incubation) were mixed with 100 mM D-glucose in PBS buffer to yield 1.0 mL. Then the samples were vortexed and snap frozen in liquid nitrogen for 2 min. After 15 minutes of thawing at 37°C the cells were centrifuged for 5 min at 2,000 g (4°C). A defined volume of each supernatant was diluted and transferred into a 96-well plate (BD falcon™ clear 96-well microtest™ plate, Franklin Lakes, NJ, USA) for subsequent photometric measurement of hemoglobin. The absorption was measured at 450 nm (Multiskan Ascent® microplate-reader, Thermo Fisher Scientific, Waltham, MA, USA). We prepared and measured each six independent samples of both incubation conditions.

### High performance liquid chromatography (HPLC)

High performance liquid chromatography was performed using a Waters HPLC (Milford, MA, USA) with a 1525 binary pump, a 717plus autosampler, a model 2487 UV/VIS dual wavelength absorbance detector set at the detection wavelength of 280 nm. Data collection and integration were accomplished using Breeze™ software version 3.30.

Method 1: The samples of the experiments elucidation the distribution of a polyphenol mixture between plasma and erythrocytes were analysed by HPLC with a combination of electrochemical and UV detection. Analysis was performed on a Zorbax SB C8 column (150 × 4.6 mm I.D., 5 μm particle size, Agilent Technologies, Palo Alto, CA, USA). Caffeic acid, M1 and (±)-taxifolin were analyzed by electrochemical detection (CLC 100; Chromsystems, Munich, Germany) using oxidation voltage of 0.5 V. Ferulic acid was analyzed by UV detection (280 nm); this detector was connected to the control system by a satellite interface (Waters). The flow rate was 1 mL/min, the injection volume 20 µL. Isocratic elution was performed using 88% aqueous phase (containing 0.6 mM 1-octanesulfonic acid sodium salt, 0.27 mM ethylenediaminetetraacetic acid disodium salt, 0.04 M triethylamine; pH 2.95 adjusted with phosphoric acid) and 12% acetonitrile. The method was validated according ICH guidelines. The method fulfilled the quality criteria for linearity, selectivity and intra- and inter-day precision.

Method 2: The samples of the experiments elucidation the uptake of M1 into human erythrocytes were analysed by HPLC with UV detection similar to the method described previously [13]. Therefore, samples were mixed with 0.6 µM p-coumaric acid as internal standard and 50 µL of 50% solution of trichloroacetic acid, vortexed for 10 s and centrifuged for 15 min at 18,000 *g* (4°C). Afterwards, 200 µL of the supernatant was immediately subjected to HPLC analysis. Separations were carried out on a SunFire® C18 column (4.6 x 150 mm; 5 µm particle size) from Waters. The mobile phase consisted of 0.2% (v/v) acetic acid and acetonitrile. Isocratic elution of M1 and internal standard was performed using 85% aqueous phase and 15% acetonitrile at a flow rate of 1.5 mL/min followed by a short flush step for eluting remaining matrix components. M1 and internal standard absorption was monitored at 280 nm. Retention time for M1 was t_R_ = 7.10±0.08 min and for internal standard p-coumaric acid t_R_ = 9.58±0.09 min. Linearity was proven between 0.15–10 µM M1 in PBS buffer (r^2^ = 0.9999; slope  = 0.2708±0.021; y-intercept  = 0.0189±0.016) analyzing five concentration levels. The lower limit of quantification for M1 in PBS buffer was 0.15 µM M1 with V_K_ (coefficients of variation) values for accuracy of 99.4% and precision of 24.3%. Interday-accuracy and –precision V_K_-values for M1 were 100.2% and 10.8% and intraday-accuracy and –precision V_K_-values comprised 96.0% and 7.9%.

### Computer-based structural comparison between glucose and M1

Calculations were made with the program SYBYL-X® (Tripos, version 1.0, August 2009). An energy field minimization was performed for the structures of glucose and M1 using the Powell method. Electrical charges and the resulting energy were calculated with MMFF94 taking various partial energies into account such as bond stretching, angle bending, torsional and Van der Waals energy. The energy-minimized molecules were used for alignments.

### Screening of erythrocyte incubation mixtures for putative M1 metabolites

About 5 mL of packed red blood cells were washed twice with a threefold volume of cold PBS buffer (8°C) centrifuged for 5 min at 952 *g* (10°C). Cells were suspended in PBS buffer to yield a cell fraction of 40%. The metabolite M1 was added to yield a concentration of 15 mM and cells were incubated for one hour at 37°C. In parallel a control was prepared containing M1 PBS buffer without erythrocytes. Cells were subsequently processed as described by Sana et al. [22]. Therefore, incubation vials were centrifuged at 1,000 *g* (4°C) and erythrocytes were lysed by addition of 150 µL cold Millipore® water. Lysates were cooled on dry ice to −25°C and 600 µL cold methanol was added. After vortexing and addition of 450 µL chloroform, samples were incubated for 30 min under frequent mixing. Another 150 µL cold Millipore® water was added and samples were frozen at −20°C for at least 8 hours. Both the upper aqueous and lower organic phase were collected and evaporated to dryness. The residue was reconstituted in 50 µL mobile phase of which 5 µL were subjected to HPLC-MS/MS analysis.

### Preparation of a M1-glutathione conjugate

Glutathione (10 µM) and the metabolite M1 (12 µM) were mixed with 1 U glutathione-S-transferase in 1 mL PBS buffer. The mixture was incubated for 30 min at 25°C. The MS/MS spectrum of the reaction product was compared with the putative glutathione adduct found in erythrocytes.

### HPLC-MS/MS conditions

High-performance liquid chromatography-MS/MS analyses were performed on an Agilent LC-MS 6460 triple-quadrupole mass spectrometer with an electrospray interface (Agilent, Böblingen, Germany). Chromatographic separations were carried out using an SunFire® C18 column (4.6 x 300 mm, 2.5 µm particle size with a guard column; Waters) at a flow rate of 0.5 mL/min using 0.1% formic acid in Millipore® water (solvent A) and acetonitrile/methanol 1:1 (solvent B) as mobile phase. A linear step gradient elution was performed: 95% to 10% solvent A in 40 min, followed by 100% B for 10 min. During screening, the electrospray interface source was operated in both the positive and negative ionization mode for later measurements of metabolites only the positive ionization mode (ESI+) was used at a capillary voltage of 3.50 kV and a desolvation temperature of 300°C. Detection was performed using multiple reaction monitoring (MRM) mode. The scan range used was 100–1000 m/z with a step size of 0.2 Da. Nitrogen was used as the desolvation and sheath gas with flow rates of 11 L/min, respectively. Nitrogen was used as the collision gas at a pressure of 45 psi. Data were analyzed using Agilent MassHunter data aquisition version B 02.01.

### Analysis of protection against oxidative damage using the AAPH assay

About 3 mL of the packed red cells were washed twice with 10 mL of the AAPH buffer and subsequently mixed with the AAPH buffer to result in a 10% (V/V) suspension. 12.0 mL buffer containing 1 µM of the metabolite M1 were mixed with 1.5 mL of the erythrocyte cell suspension and incubated under gentle shaking for 10 min at 37°C. 1.5 mL of AAPH solution (400 mM) was added either immediately or after pre-incubation of the cells with M1 for 60 min at 37°C. Subsequently samples of 800 µL were drawn and centrifuged for 2 min at 10,000 *g* at 4°C. The absorption of the supernatant was measured at 524 nm (uv-mini 1240, Shimadzu, Duisburg, Germany). For comparison a completely haemolysed sample was used. Therefore, 10 µL of the packed red cells were mixed with 990 µL of Millipore® water and subjected to one freeze-thaw cycle. The % haemolysis was calculated from the absorption of the cell supernatant in relation to the absorption of the totally haemolysed sample. The time lag for a 50% haemolysis occurred was determined.

### Statistical and data analysis

Data sets were subjected to one-way analysis of variance (ANOVA) with Bonferroni's multiple comparison test using GraphPad Prism® 4 (GraphPad Software Inc., Dan Diego, CA). Results were considered statistically significant at p≤0.05. Data are shown as mean with standard deviation (SD) or as mean and mean deviation of the mean (MDM).

## Results

### Distribution of polyphenols between human plasma and erythrocytes

**Figure 1:**
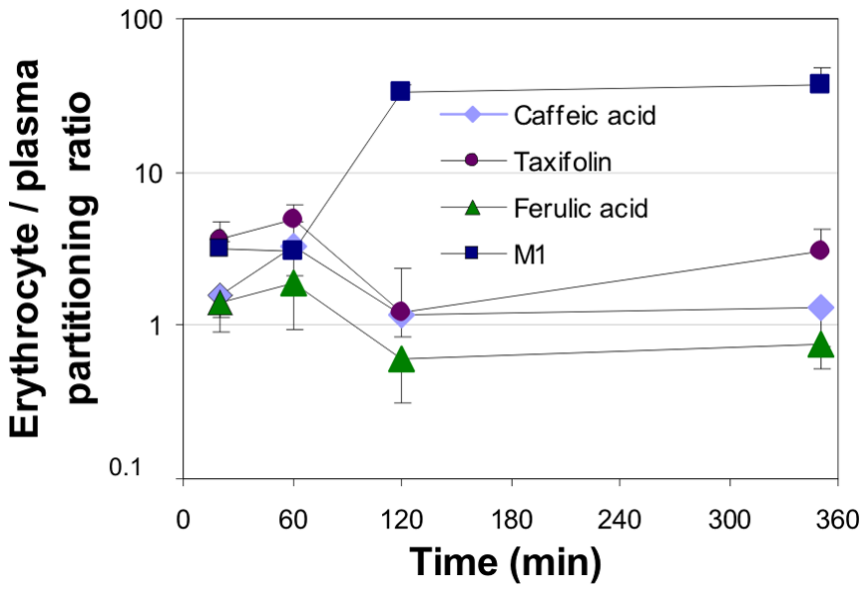
Erythrocyte/plasma partitioning ratios of polyphenols. 1.3 µM caffeic acid, 6 µM taxifolin, 80 µM ferulic acid and 6 µM of the Pycnogenol metabolite M1 were concomitantly incubated with a human blood mixture (hematocrit 0.43) at 37°C. Each data point represents the mean and standard deviation of five replicates.

The erythrocyte/plasma partitioning ratio of a mixture of caffeic acid, taxifolin, ferulic acid and the Pycnogenol® metabolite M1 (δ-(3,4-dihydroxy-phenyl)-γ-valerolactone) was determined based on a previously described method [19]. While all compounds displayed some binding to the erythrocytes after 60 min this effect was no longer pronounced after 120 min for caffeic acid, taxifolin, and ferulic acid (Figure 1). In contrast, the binding of M1 to red blood cells increased further to result in an erythrocyte/plasma partition ratio of 32.83±4.65 after 120 min and remained at 37.36±10.13 until 350 min.

**Figure 2:**
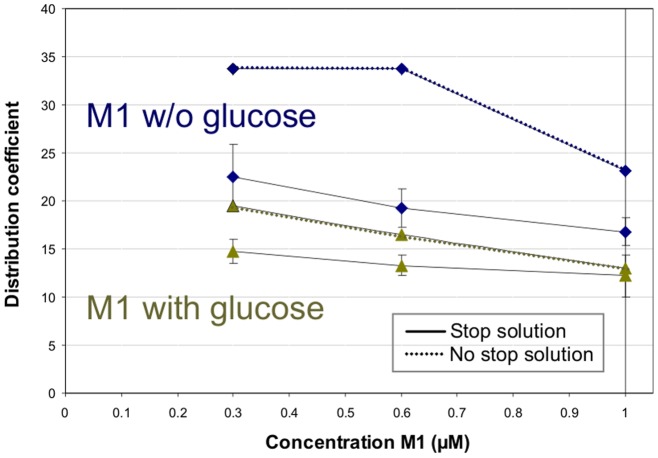
Influence of the stop solution on the uptake of M1 into human erythrocytes. In an initial experiment the distribution of different concentrations of the metabolite M1 was analyzed in the absence and presence of glucose (100 mM) with and without addition of a stop solution containing phloretin (200 µM) and cytochalasin B (20 µM). Data points of the experiments with stop solution (solid lines) represent the mean and mean deviation of the mean of three replicates, the data points without stop solution (dashed lines) were single experiments.

To elucidate whether this high partition coefficient of M1 was not only related to an adsorption to erythrocytes' outer cell membrane and diffusion processes, but to an entry and accumulation in the cells we tested the influence of various inhibitors of transporters that facilitate the uptake of small molecules into red blood cells. While no significant effects were seen with modulators of the ABCB1 (P-glycoprotein) and amino acid transporters (data not shown) a statistically significant decrease (p<0.05, one-way ANOVA with post-hoc Bonferroni test) of M1 uptake into erythrocytes was observed after 10 min in the presence of the inhibitor phloretin that e.g. inhibits the glucose transporters (GLUT-1) (Figure 2). In the presence of phloretin the erythrocyte/plasma partitioning ratio of M1 displayed a mean value of 1 while the partition coefficient increased up to 2.47±1.28 after 10 min in the absence of phloretin.

### Uptake of M1 into human erythrocytes

To elucidate whether the high partition coefficient of M1 was solely due to an adsorption to erythrocytes' outer cell membrane, diffusion processes, or the presence of other polyphenolic compounds we determined the distribution of M1 in separate experimental series. In initial experiments we analyzed the uptake of increasing concentrations of M1 (0.3 to 1 µM) into red blood cells. When we added inhibitors of glucose transporters (200 µM phloretin and 20 µM cytochalasin B) to stop a potential facilitated uptake we observed clearly reduced distribution coefficients (Figure 2). Likewise, the concomitant addition of 100 mM glucose along with M1 resulted in reduced uptake of M1. In this case, the addition of the stop solution at the end of the incubation period again reduced the distribution coefficient.

**Figure 3:**
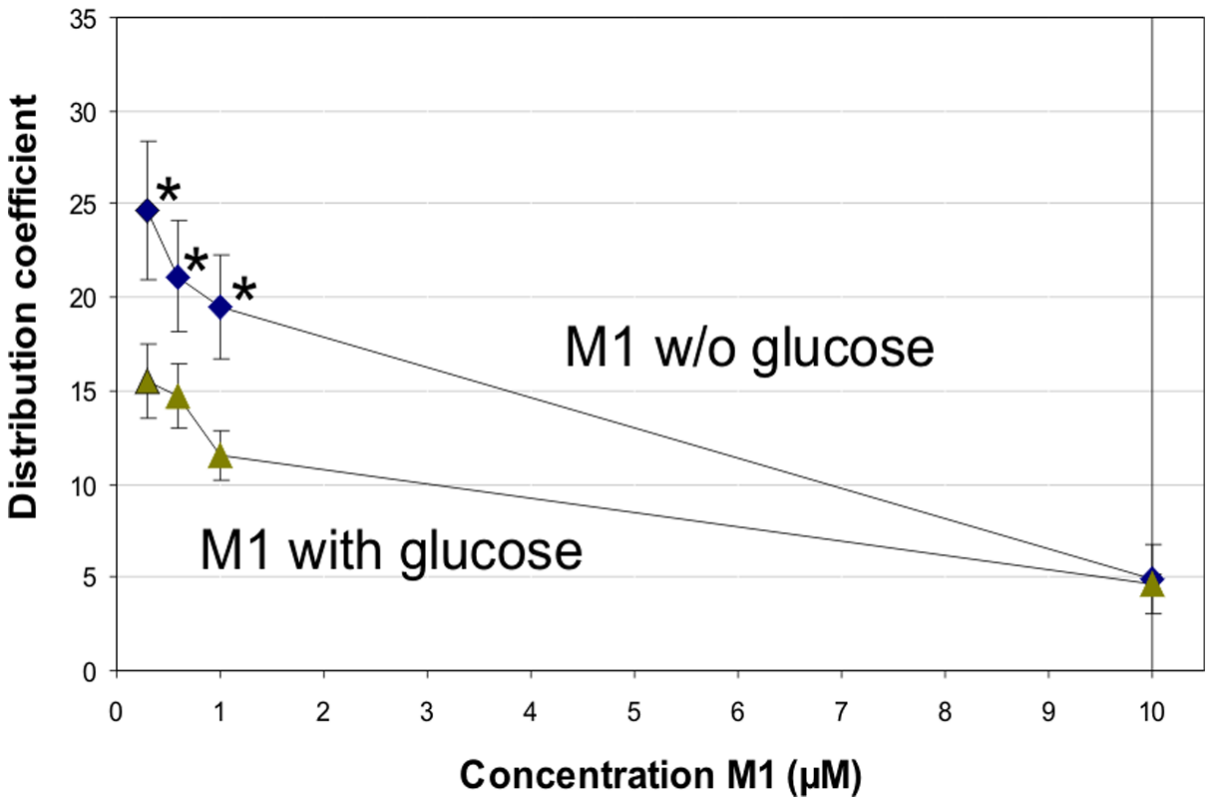
Distribution of M1 into human erythocytes. Increasing concentrations of the metabolite M1 were incubated in the absence and presence of glucose (100 mM) with human erythrocytes (hematocrit 0.043) at 4°C. The reaction was stopped after one minute with phloretin (200 µM) and cytochalasin B (20 µM). For 0.3 to 1 µM M1 the uptake into erythrocytes was statistically significant higher in absence of glucose compared to the respective uptake (0.3 to 1 µM M1) in the presence of glucose (p<0.05) and also compared to the uptake of 10 µM M1 (p<0.001; one-way ANOVA with Bonferroni post-hoc test). Each data point represents the mean and mean deviation of the mean of six replicates.

Further experiments were performed in which the stop solution containing phloretin and cytochalasin B was always added to terminate any transporter-facilitated uptake. Erythrocytes of two different individuals (blood groups A and AB, respectively) were used for the experiments. The results differed only slightly, so that the data were pooled (Figure 3). In the absence of glucose, increasing concentrations of M1 resulted in decreasing distribution coefficients, from 24.68±3.68 (0.3 µM M1) to 4.87±1.97 (10 µM M1). Thereby, the distribution coefficients determined for 0.3, 0.6 and 1 µM M1 were statistically significant higher compared to that recorded for 10 µM M1 (p<0.001; one-way ANOVA with Bonferroni post-hoc test). When 100 mM glucose was added to the red blood cells together with M1, the distribution coefficients were clearly lower, ranging from 15.48±1.96 (0.3 µM M1) to 4.66±0.57 (10 µM M1). For the concentrations of 0.3, 0.6 and 1 µM M1 the uptake into erythrocytes was statistically significant higher in absence of glucose compared to the respective M1 concentrations added simultaneously with glucose (p<0.05; one-way ANOVA with Bonferroni post-hoc test). At a concentration of 10 µM the distribution coefficient of M1 was not different in the absence or presence of glucose.

In order to exclude the possibility that the cells' exposure with high glucose concentrations altered the cell volume and thus the cell number that constituted the hematocrit, we prepared each six independent samples of both incubation conditions, lysed the erythrocytes and measured the absorption of the free hemoglobin in the supernatant (λ = 450 nm). We read absorptions of 0.8463±0.036 (n = 6; mean and SD) and 0.7983±0.083 (n = 6; mean and SD) which were not statistically significant different (p>0.05, two-sided Student's t-test).

### Structural comparison between M1 and glucose

**Figure 4:**
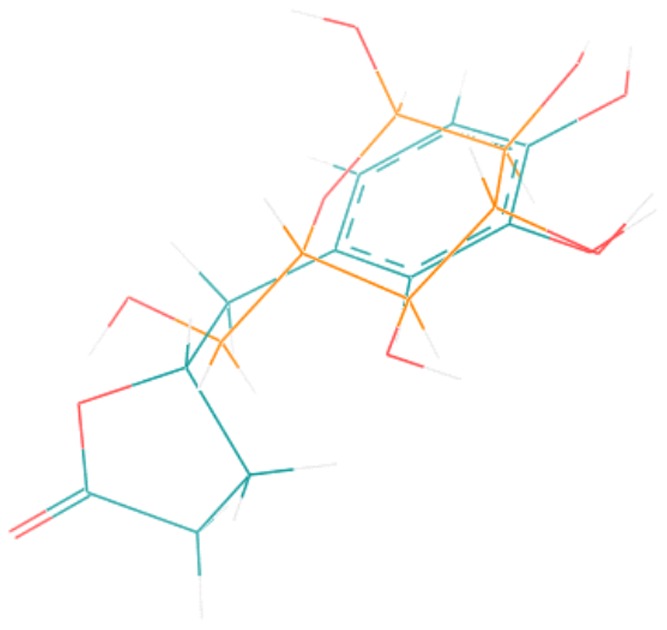
Structural alignments of M1 and glucose. The S-isomer of the metabolite M1 (δ-(3,4-dihydroxy-phenyl)-γ-valerolactone; blue) and glucose (yellow). The calculations were performed with SYBYL-X® (Tripos, version 1.0).

Structural similarities between M1 and the natural GLUT-1 substrate α-D-glucose were analysed using computer-based energy calculations. Molecule alignments showed good superimposing substructures between glucose and the S-isomer of M1 (Figure 4). The hydroxyl groups of the benzene ring of M1 aligned well with the hydroxyl function of the pyranose ring and the hydroxymethyl moiety of glucose aligned close to the lactone structure of M1. Thus, functional groups such as OH-groups that might be critical for the transport through the GLUT uptake site can adopt similar positions in the three-dimensional space. Both molecules have similar space requirements, there are no obvious steric or volume hindrances that would suggest that M1 cannot pass through the GLUT transporter.

### Screening of erythrocyte incubation mixtures for putative M1 metabolites

**Figure 5:**
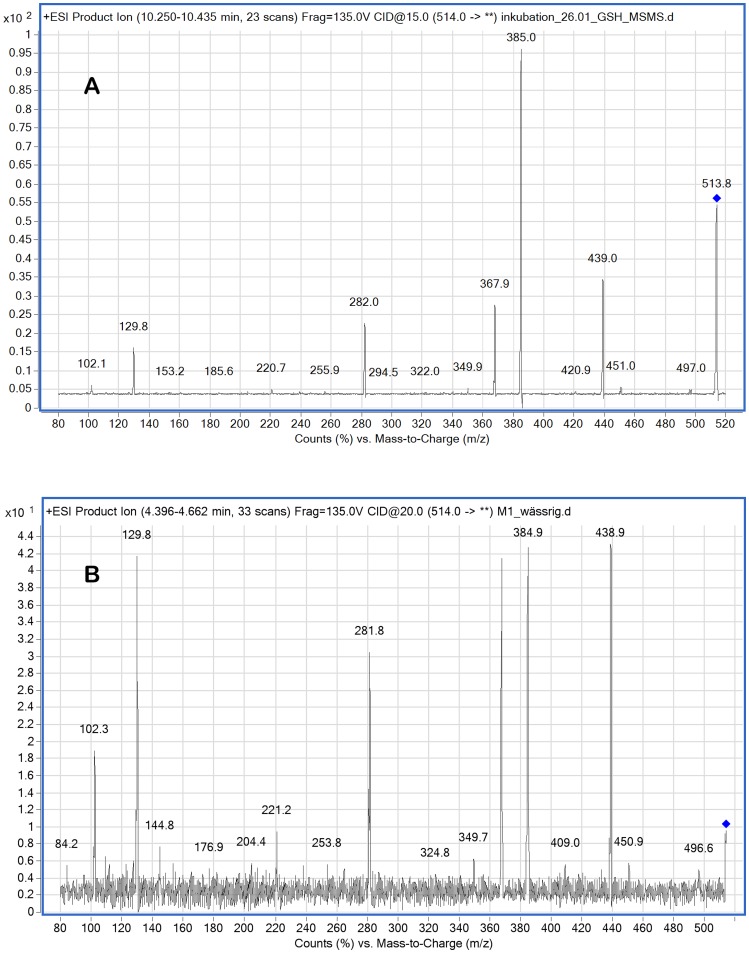
MS/MS spectra of the M1-glutathione adduct. **A**: MS/MS spectrum of the putative M1-glutathione adduct with [M+H]^+^ *m/z* of 514 found in the erythrocyte lysate after incubation with the metabolite M1. **B**: MS/MS spectrum of the M1-glutathion adduct with [M+H]^+^ *m/z* of 514 obtained after incubation of the metabolite M1 with glutathione and glutathione-S-transferase. Characteristic fragments for glutathione are pyrroglutamic acid [MH^+^-129], cysteine [MH^+^-103] and glycine [MH^+^-76] are present.

To screen for potential metabolites of M1 generated in human erythrocytes the compound was incubated with red blood cells and subjected to an extraction procedure that allowed the determination of both hydrophilic and lipophilic metabolites [22]. The extracts were scanned by LC-MS/MS in both the positive and negative ionisation mode over a range of 100–1000 *m/z* with a step size of 0.2 Da. For comparison an erythrocyte extract that was not exposed to M1 was used. During this screening procedure a new signal with [M+H]^+^ *m/z* of 514 was detected (Figure 5, A). This molecular mass was consisted with a glutathione adduct of M1. To obtain a reference compound M1 and glutathione were incubated in the presence of glutathione-S-transferase and the resulting MS/MS spectrum of the reaction product was analysed (Figure 5, B). Besides the signal with [M+H]^+^ *m/z* of 514 fragments described to be characteristic for glutathione such as pyrroglutamic acid [MH^+^-129], cysteine [MH^+^-103] and glycine [MH^+^-76] [23], [24] were detectable.

### Analysis of protection against oxidative damage using the AAPH assay

**Figure 6:**
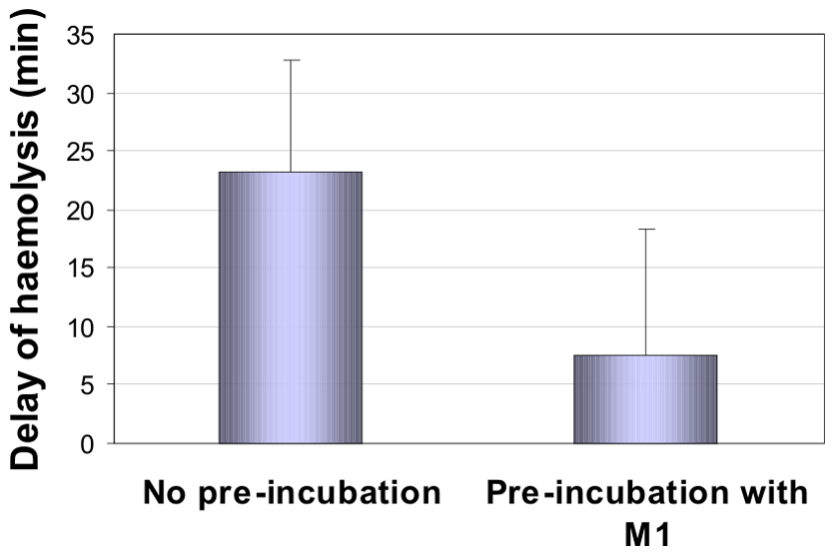
Protection of erythrocytes against oxidative haemolysis in the presence of M1. Haemolysis of a 1% human erythrocytes suspension in the presence of the metabolite M1 (1 µM) was determined in an AAPH-assay. Erythrocytes were either co-incubated with M1 (left column) or pre-incubated with M1 for 60 min (right column), and delay of haemolysis was determined with reference to an incubation mixture without addition of M1. Columns represent the mean and standard deviation of three replicates.

To elucidate whether the red blood cell bound M1 or its glutathione adduct conferred a different degree of the erythrocytes' protection against oxidative damage an AAPH assay was performed. Therefore, erythrocytes M1 was either directly added to the incubation mixture or pre-incubated with the red blood cells for 60 min to allow for M1 uptake and metabolism. Subsequently the delay of 50% haemolysis was determined with reference to an incubation mixture without addition of M1 (Figure 6). The more pronounced delay of induced haemolysis was seen when M1 was freshly added to the incubation mixture (Δt of 23.1±9.6 min) compared the pre-incubation conditions (Δt of 7.47±10.8 min).

## Discussion

In the present investigation we analyzed the distribution of polyphenols into human red blood cells and found a strong indication for a facilitated uptake and accumulation of the Pycnogenol metabolite δ-(3,4-dihydroxy-phenyl)-γ-valerolactone (M1) in erythrocytes. The partitioning of M1 into erythrocytes was significantly diminished at higher concentrations of M1, in the presence of glucose and upon the addition of a transporter-inhibiting stop solution containing phloretin and cytochalasin B. This is suggestive of a facilitated uptake of M1 into red blood cells, possibly via GLUT-1. This notion was further supported by structural similarities between the natural GLUT-1 substrate α-D-glucose and the S-isomer of M1. Erythrocytes metabolize M1 to form a novel glutathione adduct which role needs to be further investigated.

Many plant extracts used as phytotherapeutics or dietary supplements exhibit bioactivity [4], [25] while plasma concentrations of individual compounds are typically in the nanomolar range [5], [6]. However, these low concentrations are usually not sufficient to exert any measurable activity in *in vitro* cell culture assays [11], [26]. It is possible that either the compounds detected in plasma are not the key effectors of bioactivity or that measurable concentrations also reside in compartments other than the plasma. It has been shown that the recoveries of resveratrol and quercetin were significantly higher from whole blood compared to plasma [12]. We recently found a pronounced binding of M1 to endothelial cells and monocytes/macrophages which was decreased in the presence of phloretin, suggesting a facilitated uptake [13].

Red blood cells represent more than 99% of the total cellular space of human blood and can thus constitute a significant compartment for distribution. Various drugs and endogenous compounds bind to erythrocytes [15]. Red blood cells were shown to bind polyphenols, and gallic acid, curcumin and resveratrol were most extensively bound [27]. Erythrocyte/plasma partitioning ratios higher than 0.25 indicate association of the respective compound with red blood cells, which could be either an uptake into the cells or binding to the surface membranes [15]. In our experiments with a polyphenol mixture all compounds revealed higher red blood cell/plasma partitioning ratios than 1.0 up to 60 min. Afterwards the partition coefficients of caffeic acid, taxifolin and ferulic acid decreased. In contrast, the erythrocyte/plasma partitioning ratio of M1 increased further to over 30 remained at that high level up to 350 min. This is suggestive of an accumulation of M1 within or on the surface of red blood cells.

It has been discussed that the compound's lipophilicity is a major determinant for its distribution in the body [15]. Indeed, in an analysis of whole blood compared to analysis of plasma it appeared that the more lipophilic resveratrol was bound to a higher extent to blood cells compared to quercetin [12]. However, in the present study we did not observe any correlation between the compounds' lipophilicity and the binding to erythrocytes, just as we previously did not find a correlation of the polyphenols' lipophilicity or topological polar surface area with plasma protein binding or nonspecific binding to material surfaces [16]. Especially the high binding of M1 to erythrocytes was striking since its plasma protein binding is significantly lower compared to caffeic acid, taxifolin and ferulic acid. We suspected that the accumulation of M1 in erythrocytes was not solely driven by diffusion processes.

When we determined the binding of M1 alone we found high uptake into human red blood cells already after one minute and a statistically significant decrease of the distribution coefficient with increasing concentrations. The simultaneous addition of M1 and glucose to erythrocytes significantly reduced the uptake of M1 at lower concentrations (0.3–1 µM), but no further decrease was seen at the highest tested concentration of 10 µM M1. These results are consistent with a transporter-facilitated uptake and a substrate inhibition at higher M1 concentrations. Since erythrocyte glucose transport is facilitated by GLUT-1 transporter which is highly expressed in these cells, accounting for 10% of the total protein mass [28], [29], it appears most likely that M1 is taken up via this transport system as well. Another indication for this notion is that the addition of a stop solution containing phloretin and cytochalasin B at the end of the incubation period clearly reduced the distribution coefficient of M1. Both phloretin and cytochalasin B are inhibitors of GLUT-1 transporters [30] although they are not highly selective. Phloretin for example interacts with various transporters such the monocarboxylate transporter [31], sodium glucose co-transporter SGLT-1 [32], volume-sensitive chloride channels [33], aquaporin water channels [34] or the red blood cell urea transporter [35]. Though phloretin also binds to other GLUT isoforms [36] it potently inhibits the GLUT-1-type glucose transporter [37].

Besides the facilitating the uptake of glucose into red blood cells GLUT-1 also transports other molecules such as galactose, mannose, L-dehydroascorbic acid (DHA) and tyrosine [38]–[40]. Interestingly, compounds such as DHA can be taken up into human erythrocytes although they are present at a significantly lower concentration in plasma compared to glucose. It has been suggested that the GLUT-1 uptake profile might be modulated by GLUT binding partners such as stomatin [41], [42]. Association of GLUT-1 with stomatin was shown to decrease glucose uptake and enhance DHA uptake [41]–[43]. While we did not investigate any mechanistic background we also observed that M1 was taken up by human erythrocytes in the presence of an excess concentration of glucose.

Recently docking studies have shown that besides α-D-glucose also quercetin might slide through the GLUT-1 transporter [44], thus suggesting that this transporter accepts structurally variable molecules. Structural comparisons between α-D-glucose and the S-isomer of M1 revealed good alignment which further supports the notion that a facilitated uptake of M1 into erythrocytes might be possible since there are no obvious structural restrictions that make it unlikely that M1 can pass through the GLUT transporter. So far it is not clear yet which M1 isomer predominantly occurs *in vivo*. Though a preferred excretion of one isomer has been described [9], [10] the designation as “–” isomer does not allow to deduce whether this is the R- or S-isomer according to CIP nomenclature.

The significance of partitioning of drugs into red blood cells has been detailed earlier [14], [15]. The distribution into erythrocytes contributes to the storage, transport and metabolism of molecules and may affect their activity [45]. The elimination half-life of compounds from different blood constituents might vary, the discharge from erythrocytes is often faster than the loss from plasma proteins so that red blood cells constitute a transport system with high capacity and low affinity compared to plasma proteins [14]. However, it is also known that the half-life of a compound can be longer in erythrocytes compared to the plasma half-life, e.g. for methotrexate [45].

Due to an enhanced uptake of M1 into red blood cells the total presence of this compound *in vivo* might be overall higher than previously deduced from its plasma concentrations [6]. It can be speculated that an enhanced uptake of M1 will also seen in other tissues that express GLUT-1, such as the blood-brain barrier [46]. Furthermore it is possible that the transport in or on red blood cells facilitates an efficient exchange of the compound between the erythrocyte and the capillary endothelium [14].

After partitioning into red blood cells compounds might be subjected to intracellular metabolism. This has been described for many drugs and also for endogenous molecules [15], [45]. Thus, after observing an accumulation of M1 in human erythrocytes we screened the cell lysates for potential metabolites and identified a M1 glutathione conjugate. Red blood cells contain 200–400 µg glutathione per mL blood [47] and possess a glutathione-S-transferase [48]. Formation of glutathione adducts has been described as part of detoxification of xenobiotics [49]. Recently it has been described that glutathione adducts with flavonoids, e.g. quercetin, are formed after scavenging of free radicals and formation of electrophilic quinones [50], [51]. M1 also displays structural features that allow oxidation under formation of an electrophilic benzoquinone that would be preferentially attacked at C4 by the nucleophilic thiol moiety of glutathione. However, this is not supported by the MS/MS spectrum of the M1-glutathione adduct with [M+H]^+^ *m/z* of 514 which is not consistent with formation of a quinone. Glutathione conjugation is a reversible process for certain compounds, e.g. for quercetin [50], [52], [53]. However, we did not investigate whether the M1 adduct formation is a reversible process and the precise role of the glutathione conjugate still needs to be clarified.

Quercetin and other polyphenols were reported to inhibit oxidative haemolysis of red blood cells [27], [54]. We previously demonstrated in various assays that the Pycnogenol metabolite M1 is a potent radical scavenger [11]. We now analysed whether a one hour pre-incubation and thus accumulation and conjugate formation of M1 in erythrocytes changed the resistibility of the cells against oxidative haemolysis. The protection against haemolysis was less pronounced after pre-incubation compared to direct addition of M1 to the erythrocyte incubation mixture. It can be concluded that M1 confers protection against oxidative stress primarily if present outside the cell. This is consistent with the results of Koren et al. [27] who found that the polyphenols bound the erythrocytes' surface form antioxidant depots and protect against oxidative stress.

Our study has a number of limitations. The initial experiments were done with mixtures of all polyphenols and it is possible that the partitioning behaviour of individual compounds influenced the partitioning of others, e.g. by inhibiting a relevant transporter system. However, we think that the significant decrease of M1 uptake into erythrocytes at higher concentrations of this metabolite as well as in the presence of glucose support our notion of an enhanced uptake of M1 into red blood cells. The intracellular presence of M1 was also confirmed by the detection of a glutathione conjugate. We did not elucidate the extent of glutathione adduct formation compared to M1 uptake into red blood cells or whether an enhanced outward transport of the glutathione conjugate or a reverse of the conjugation reaction occurred. Thus, we do not know whether the presence of M1 in erythrocytes is altered due to its metabolism. Finally, it is possible that M1 is taken up into erythrocytes by a transporter other than GLUT-1. However, the high abundance of GLUT-1 transporters in red blood cells [28], [29] and the structural similarity of M1 and the natural GLUT-1 substrate glucose suggest an involvement of GLUT-1. Yet it cannot be excluded that additional diffusion processes play a role as it was suggested by Sugano et al. that passive and carrier-mediated processes can coexist [55]. Finally, we did not investigate whether the glucose flux in erythrocytes was influenced by M1 or the precise type of interaction with the GLUT-1 transporter. Kinetic and mechanistic details of the erythrocyte glucose transport are still ascertained [21], [56]. While GLUT-1 has binding sites for polyphenols such as quercetin or phloretin the type of interaction with the transporter appears to be complex as compounds can behave as competitive or noncompetitive inhibitors regarding glucose uptake or exit [30]. Though we do not provide further details on the transport of M1 we uncovered a novel disposition site for this bioactive compound of plant origin.

To summarize, we found that caffeic acid, taxifolin, ferulic acid, and M1 all bind to human erythrocytes, but only the Pycnogenol metabolite M1 revealed accumulation within the cells. The more than 30-fold increase in the erythrocyte/plasma partitioning ratio indicates that red blood cells are a significant compartment for distribution of M1. M1 was previously shown to exert pronounced anti-inflammatory activities [11], but the plasma concentrations were rather low in the nanomolar range [6]. Our present results thus substantiate that low plasma concentrations do not necessarily reflect low presence of the compound *in vivo*. The uptake of M1 into erythrocytes was diminished in the presence of glucose and at higher concentrations of metabolite itself, suggesting a facilitated uptake of M1 into red blood cells, possibly via GLUT-1. In erythrocytes an intracellular conjugation of M1 yielding a glutathione adduct was detected, but the precise role of the reaction needs to be further investigated. Thus, we present novel data on the disposition of the bioactive maritime pine bark extract metabolite M1. This might help to further understand the *in vivo* behaviour of plant extract components.
